# Identification of Small Molecules that Disrupt Signaling between ABL and Its Positive Regulator RIN1

**DOI:** 10.1371/journal.pone.0121833

**Published:** 2015-03-26

**Authors:** Pamela Y. Ting, Robert Damoiseaux, Björn Titz, Kenneth A. Bradley, Thomas G. Graeber, Virneliz Fernández-Vega, Thomas D. Bannister, Peter Chase, Reji Nair, Louis Scampavia, Peter Hodder, Timothy P. Spicer, John Colicelli

**Affiliations:** 1 Molecular Biology Institute, Jonsson Comprehensive Cancer Center, Department of Biological Chemistry, David Geffen School of Medicine, University of California Los Angeles, Los Angeles, California, United States of America; 2 California NanoSystems Institute, University of California Los Angeles, Los Angeles, California, United States of America; 3 Department of Molecular and Medical Pharmacology, Crump Institute for Molecular Imaging, University of California at Los Angeles Metabolomics and Proteomics Center, California NanoSystems Institute and Jonsson Comprehensive Cancer Center, University of California Los Angeles, Los Angeles, California, United States of America; 4 Department of Microbiology, Immunology and Molecular Genetics, Jonsson Comprehensive Cancer Center, and California NanoSystems Institute, University of California Los Angeles, Los Angeles, California, United States of America; 5 The Scripps Research Institute-FL, Lead Identification, Translational Research Institute, Jupiter, Florida, United States of America; 6 The Scripps Research Institute-FL, Department of Chemistry, Translational Research Institute, Jupiter, Florida, United States of America; University of Pittsburgh School of Medicine, UNITED STATES

## Abstract

Constitutively active BCR-ABL kinase fusions are causative mutations in the pathogenesis of hematopoietic neoplasias including chronic myelogenous leukemia (CML). Although these fusions have been successfully targeted with kinase inhibitors, drug-resistance and relapse continue to limit long-term survival, highlighting the need for continued innovative drug discovery. We developed a time-resolved Förster resonance energy transfer (TR-FRET) -based assay to identify compounds that disrupt stimulation of the ABL kinase by blocking its ability to bind the positive regulator RIN1. This assay was used in a high throughput screen (HTS) of two small molecule libraries totaling 444,743 compounds. 708 confirmed hits were counter-screened to eliminate off-target inhibitors and reanalyzed to prioritize compounds with IC_50_ values below 10 μM. The CML cell line K562 was then used to identify five compounds that decrease MAPK1/3 phosphorylation, which we determined to be an indicator of RIN1-dependent ABL signaling. One of these compounds is a thiadiazole, and the other four are structurally related acyl piperidine amides. Notably, these five compounds lower cellular BCR-ABL1 kinase activity by blocking a positive regulatory interaction rather than directly inhibiting ABL catalytic function.

## Introduction

Chromosome translocations that create ABL kinase fusion proteins are responsible for 95% of chronic myelogenous leukemia (CML), as well as some cases of acute lymphoblastic leukemia (ALL) and acute myelogenous leukemia [1]. The most common translocation fuses BCR on chromosome 22 to ABL1 on chromosome 9 [2], creating a constitutively active BCR-ABL1 kinase that promotes hyperproliferation of progenitor hematopoietic cells. The selective kinase inhibitor imatinib has been successful in achieving what appear to be complete cytogenetic responses in most CML patients [3]. Treatment is not curative, however, because dormant cancer cells can develop resistance to imatinib through mutations in BCR-ABL1 [4,5]. The rate of patient relapse is 18% after a median of five years of kinase inhibitor therapy [6]. The most refractory mutation, BCR-ABL1^T315I^, is not responsive to the second generation kinase inhibitors nilotinib [7], dasatinib [8] and bosutinib [9]. Although the third generation kinase inhibitor ponatinib is effective against BCR-ABL^T315I^ [10], compound mutations still lead to resistance in some patients [11,12].

The constitutive activity of BCR-ABL1 is attributed to loss of the ABL1 amino terminal autoinhibitory peptide, which is typically myristoylated [13,14], and its replacement by a BCR-encoded oligomerization domain [15]. However, BCR-ABL1 retains the autoinhibitory ABL-SH2 and SH3 domains common in non-receptor tyrosine kinases [16]. RIN1 stimulates ABL catalytic activity by directly binding these domains and relieving their autoinhibitory effect on the kinase domain [17–19]. Retention of ABL-SH2 and SH3 sequences in BCR-ABL1 suggests that, although constitutively active relative to normal ABL kinases, BCR-ABL1 is still subject to positive regulation by RIN1. Indeed, altered RIN1 expression correlates directly with BCR-ABL1 activity [20].

RIN1 binding to ABL proteins is initiated by a low affinity interaction between a proline rich motif on RIN1 and the SH3 domain of ABL [17]. ABL subsequently phosphorylates RIN1 on Y^36^, which then binds to the SH2 domain of ABL. This leads to a stable divalent interaction between the proteins and alleviation of ABL autoinhibition [18]. RIN1 co-localizes with BCR-ABL1 when exogenously expressed in Cos-7 cells [21]. In addition, RIN1 binds to and enhances the leukemogenic properties of BCR-ABL1 [18,20] and RIN1 is required for *ex vivo* BCR-ABL1 transformation of bone marrow cells to a state of growth factor independence. Moreover, RIN1 depletion in the ALL cell line TOM-1 increased imatinib sensitivity. This is consistent with RIN1 functioning as a BCR-ABL1 stimulator that works allosterically to promote catalytic activity. Notably, imatinib-resistant primary ALL cells from a BCR-ABL1^T315I^-relapsed patient were re-sensitized to imatinib by RIN1 silencing [20].

To identify a novel class of drugs that exploits ABL’s reliance on RIN1 for full kinase activity, we developed a time-resolved Förster resonance energy transfer (TR-FRET) high throughput screen (HTS) that provides an indirect measure of RIN1 binding to ABL. Compounds that block RIN1::ABL association might be effective as inhibitors of BCR-ABL1 mutants that are resistant to catalytic site inhibitors, as components in multi-domain targeting treatments, and as molecular probes to further study the mechanism of RIN1-induced ABL stimulation. We screened a combined 444,743 compounds at the UCLA Molecular Shared Screening Resource (MSSR) and The Scripps Research Institute, Florida (TSRI). The screen identified five compounds of interest that disrupt RIN1-stimulated BCR-ABL1 signaling in the CML cell line K562.

## Results

### Assay development and validation

**Fig 1:**
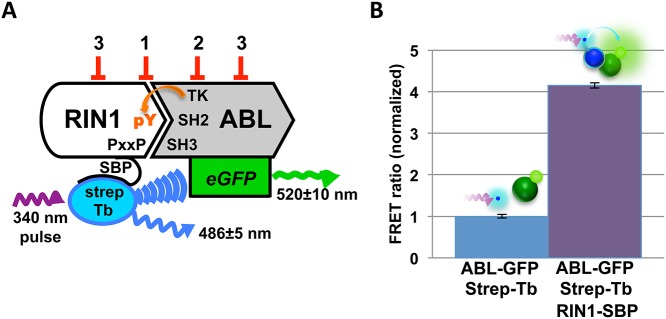
TR-FRET screen for RIN1::ABL1 interaction inhibitors. (**A**) Binding between RIN1 and ABL is initiated by a proline rich motif in RIN1 binding to the ABL-SH3 domain. ABL phosphorylates RIN1-Y^36^, which then binds the ABL-SH2 domain. For the assay, ABL was fused to eGFP and RIN1 was fused to a streptavidin binding peptide (SBP) that connects to a streptavidin-terbium complex. A 340 nm pulse excites terbium, which can transfer energy to excite the GFP acceptor if the fluorophores are in close proximity, reflecting RIN1::ABL binding. Predicted FRET inhibitor classes: 1. Orthosteric inhibitors, 2. Direct ABL kinase inhibitors and 3. Allosteric inhibitors. (**B**) RIN1::ABL binding was quantified as a FRET ratio: GFP emission at 520 nm to terbium emission at 486 nm. The negative control was donor and acceptor fluorophores only (no RIN1) and was normalized to 1.

To measure binding between purified RIN1 and ABL proteins we designed a quantitative TR-FRET based assay. The first assay component is full-length human RIN1 fused at the carboxy terminus to a streptavidin binding peptide (RIN1-SBP), which binds stably to a streptavidin-terbium complex that serves as the TR-FRET donor. The second assay component is the amino terminal half of ABL1 (includes the SH3, SH2 and tyrosine kinase domains and is fully activatable by RIN1) fused to eGFP (ABL1-eGFP), which serves as the TR-FRET acceptor (Fig 1A). Because the lanthanide ion donor, terbium, exhibits slowly decaying luminescence [22], FRET emission can be detected after excitation pulse termination, reducing background relative to signal.

The assay readout was calculated as a ratio of donor and acceptor emissions, a measurement independent of donor and acceptor concentrations that allows for reliable comparison across assays. Assay components were mixed in kinase buffer and incubated for one hour at room temperature to allow for RIN1-Y^36^ phosphorylation by ABL1 and subsequent formation of a stable divalent interaction between these proteins. Each multi-well assay plate included negative control samples (streptavidin-terbium, ABL-eGFP and ATP *without* RIN1-SBP). Positive control samples (all assay components included) gave robust and reproducible increases in the FRET ratio following incubation, reflecting the interaction of RIN1 and ABL1 constructs and subsequent energy transfer from terbium to eGFP (Fig 1B).

**Fig 2:**
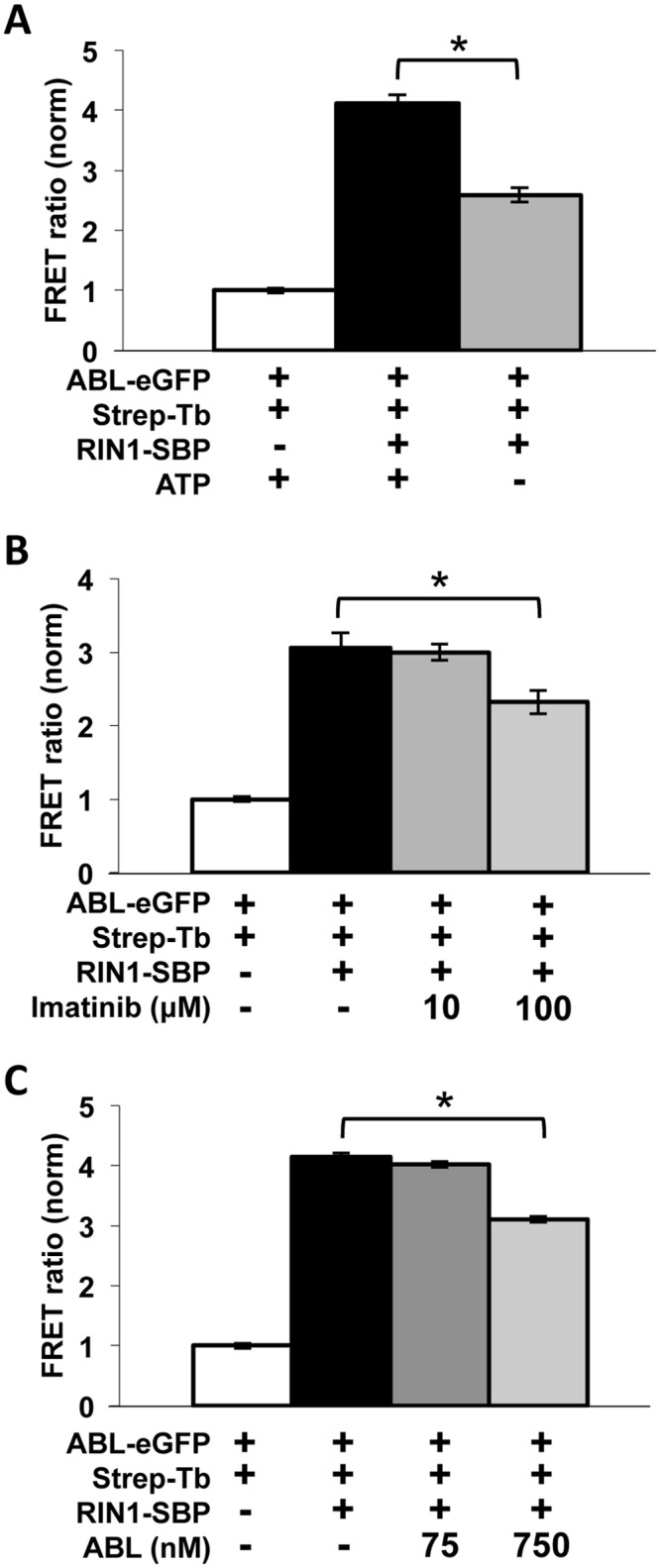
Validation of TR-FRET screening assay for detection of RIN1::ABL binding. Binding was quantified as a ratio of GFP emission at 520 nm to terbium emission at 486 nm. The negative control was normalized to 1. Experiments were performed in quadruplicate. (**A**) ATP was omitted from the buffer mix to prevent RIN1 phosphorylation by ABL. *p = 3.0x10^-6^ (**B**) 10 μM or 100 μM imatinib was added to inhibit ABL kinase activity. *p = 0.001 (**C**) Added untagged ABL competed with ABL-eGFP (1:1 and 10:1 ratios were used). *p = 1.4x10^-7^

The assay was validated using several additional controls. Omission of ATP significantly decreased the FRET ratio (Fig 2A), reflecting the loss of ABL-mediated RIN1-Y^36^ phosphorylation and resultant loss of ABL-SH2 domain binding. Addition of the ABL kinase inhibitor imatinib also decreased the FRET ratio (Fig 2B), as expected if RIN1 tyrosine phosphorylation were blocked. The remaining signal in both cases is likely due to low affinity binding between the proline-rich motif of RIN1 and the ABL-SH3 domain. Addition of untagged ABL1 into the assay also decreased the FRET ratio in a dose-dependent manner due to competition with the ABL1-eGFP FRET acceptor construct for binding to RIN1 (Fig 2C). The Z’-factor of the assay was 0.97±0.03, indicating a high degree of assay sensitivity and reproducibility for high throughput screening.

Based on the assay design and control results, we anticipated identifying three types of inhibitors: 1) compounds that work through steric interference by binding directly to residues at the protein-protein interface, 2) kinase inhibitor compounds that decrease ABL-mediated phosphorylation of RIN1-Y^36^ and 3) compounds that work allosterically on one protein to alter conformation and diminish interaction with its partner protein (Fig 1A).

### High-throughput screening and counter-screening

**Fig 3:**
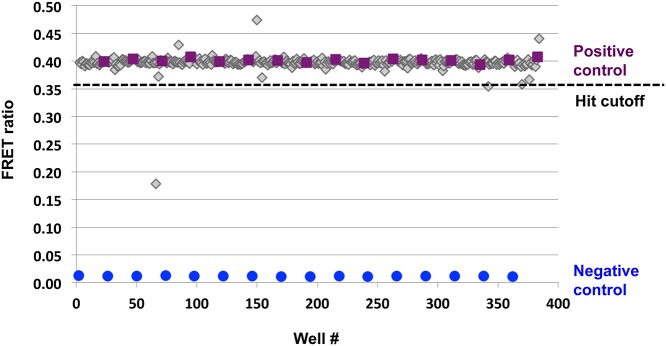
Representative data from primary HTS. Example of 384-well plate data presented as a scatterplot. Each plate was screened with positive (purple) and negative (blue) controls consisting of streptavidin terbium and ABL-eGFP with and without RIN1-SBP, respectively. Screening compounds are represented in gray. The hit cutoff was a plate-based 3-standard deviation decrease in the FRET ratio.

High-throughput screens were carried out at two facilities. At the UCLA Molecular Screening Shared Resource 86,259 compounds were screened at a concentration of 10 μM, with an average Z-factor of 0.64. At the Scripps Research Institute, Florida, 358,484 compounds from the NIH MLSMR small molecule library were screened at 7.35 μM, with an average Z-factor of 0.76 (Table 1). Z’-factor was used to evaluate plate data quality without the interference of test compounds. Individual plate-based 3-standard deviation decreases from the mean FRET ratio (Z score ≤ -3) were used to select hits on a plate per plate basis, with the majority of compounds having no effect in the assay (Fig 3). The combined hit rate was 0.36%. In total, 1,637 hits were identified and 708 of these were confirmed by testing in triplicate (Table 1).

**Table 1:** High throughput screen results.

		Molecular Screening Shared Resource (UCLA)	The Scripps Research Institute, Florida
**Primary screen**	**Compounds tested**	86,259 compounds at 10 μM	358,484 compounds at 7.35 μM
	**Avg. Z’-factor**	0.94 ±. 03^a^	0.90 ±. 01^b^
	**Avg. Z-factor**	0.64 ± 0.38^a^	0.76 ± 0.38^b^
	**hits** ^c^	202 hits = 0.2%	1435 hits = 0.4%
**Confirmation and counter- screens**	**confirmed**	78 confirmed	630 confirmed
	**quenchers**	(7 quenchers)	(93 quenchers)
	**Biotin analogs**	(4 biotin analogs)	(11 biotin analogs)
	**IC**_**50**_ **< 10 μM**	11 active	82 active
	**Selected for further analysis**	3 compounds	21 compounds

^a^ n = 254 plates

^b^ n = 296 plates

^c^ hits were identified by Z-score ≤-3 computed on a per plate basis.

**Fig 4:**
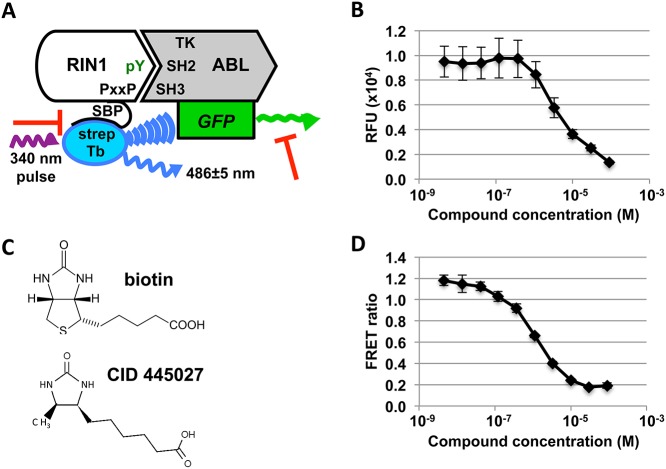
Elimination of off-target inhibitors. (**A**) Two types of off-target inhibitors were predicted (red T-bars): compounds that quench GFP fluorescence, and biotin-like compounds that bind and sequester streptavidin. (**B**) Hit compounds were tested in triplicate dose-response curves with ABL-eGFP. Concentration response curve data from a representative GFP quencher (methyl 3,4,6-trihydroxy-5-oxo-5H-benzo[7]annulene-8-carboxylate) is shown. (**C**) Biotin mimics were identified by *in silico* screening for structural similarity to biotin. (**D**) Hit compounds were re-tested in the TR-FRET assay in triplicate 10-point dose-response curves to prioritize those with IC_50_<10 μM. Data from the representative compound CID 24512426 is shown.

Based on the assay design, two types of false positive inhibitors were predicted: compounds that quench GFP fluorescence, and compounds that bind with high affinity to streptavidin and sequester it from RIN1-SBP (Fig 4A). We identified GFP quenchers by incubating the compounds with ABL-eGFP and measuring GFP fluorescence (Fig 4B). Compounds were tested in triplicate dose-response curves and quenchers were defined as compounds that decreased GFP fluorescence by ≥ 50% at 10 μM. To eliminate compounds that might bind with high affinity to streptavidin and sequester it from RIN1-SBP, confirmed hits were examined for structural similarity to biotin (Fig 4C). Lastly, we narrowed the list of hits by performing concentration-response curves in triplicate and giving higher preference to compounds with IC_50_ <10 μM (Fig 4D). Confirmed and targeted hits from the UCLA MSSR screen can be found in S1 Table, and hits from the TSRI screen at PubChem BioAssay AID 602181 (https://pubchem.ncbi.nlm.nih.gov/assay/assay.cgi?aid=602181).

**Fig 5:**
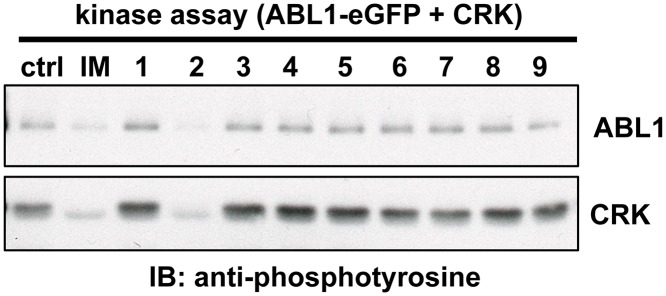
Theaflavin-3,3’-digallate is a direct ABL kinase inhibitor. 0.5 nM purified ABL-eGFP was mixed with 2 μM CRK and 10 μM test compound and incubated in kinase buffer for 30 minutes at 30°C. Samples were analyzed by immunoblot using anti-phosphotyrosine. All the hits were screened, and a representative immunoblot is shown here. Test compounds were as follows: DMSO (ctrl), imatinib (IM), CID 16312764 (1), CID 467320 (2), CID 7457610 (3), CID 6084 (4), CID 4844281 (5), IUPAC 1-(4-phenylphenyl)-2-[4-(pyrimidin-2-yl)piperazin-1-yl]ethan-1-ol (6), CID 9291491 (7), CID 3991439 (8) and CID 16350799 (9).

Although the objective of our TR-FRET assay was to identify a unique class of compounds that disrupt the RIN1::ABL interaction allosterically or by binding to the protein-protein interface, compounds that directly inhibit ABL catalytic activity could also appear as hits (Fig 1A). To identify these direct ABL kinase inhibitors, we screened the compounds using an *in vitro* kinase assay. Purified ABL1-eGFP was incubated with CRK, a well-characterized substrate, in kinase buffer containing ATP. CRK and ABL phosphorylation were detected by anti-phosphotyrosine immunoblot, and both were reduced by imatinib. All of the hits were screened, and a representative immunoblot is shown in Fig 5. Using this assay we identified one hit compound, theaflavin-3,3’-digallate, that directly inhibits ABL trans-phosphorylation of CRK as well as ABL (Fig 5). Although this tea polyphenol has been studied for its antioxidant [23] and anti-cancer effects [24,25], this is the first report of its activity as an ABL kinase inhibitor.

After eliminating off-target inhibitors, the best remaining hits clustered into 7 scaffold groups. Pyridone/pyrimidines, acyl piperidine carboxamides and heterocyclic amides are the largest clusters, with 8, 9 and 17 members, respectively. In addition, we identified three macrocycles, four tetrazoles, one indole alkaloid and one thiadiazole (S1 Fig). We selected 21 hits for further analysis (S2 Table) based on several criteria: 1) strong inhibition in the TR-FRET assay (IC_50_ <12 µM); 2) high maximal inhibition (>60%); 3) low or moderate hit rate of compounds in other assays reported in PubChem (<10%); 4) chemical tractability; and 5) positive evidence for structure activity relationships in hit scaffolds.

### Five hit compounds decrease MAPK1 phosphorylation

To independently confirm that the hit compounds inhibit RIN1::ABL binding, and to identify those that effectively block signal transduction in cells, we developed a secondary assay to measure the effect of the compounds in the CML cell line K562. We sought to employ ABL substrate phosphorylation as a read-out of RIN1-dependent ABL signaling because of its suitability for cell-based screening. To identify RIN1-dependent ABL substrates, we employed immunoaffinity purification of tyrosine phosphopeptides followed by tandem mass spectrometry to generate phosphotyrosine profiles of previously described K562 cells expressing a control shRNA or a RIN1-targeted shRNA [20]. We noted downregulation of many known ABL substrates such as BCR pY^644^, ABL1 pY^253^, ABL1 pY^257^ and ABI1 pY^213^, but one of the most robust losses of phosphorylation was seen for MAPK1 pY^187^ (S3 Table).

**Fig 6:**
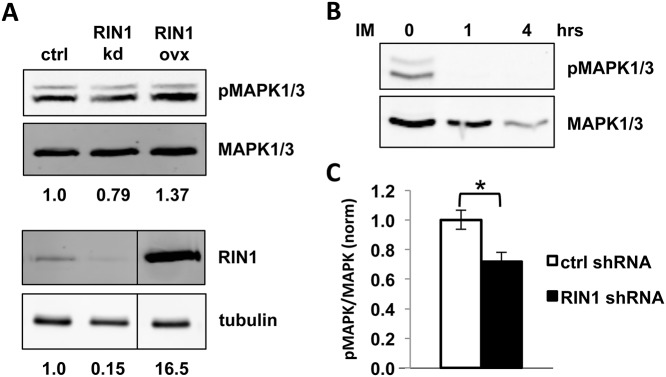
MAPK1/3 phosphorylation is RIN1 and BCR-ABL1-dependent in K562 cells. (**A**) K562 cells expressing a control vector, RIN1 shRNA or RIN1 over-expression construct were analyzed by immunoblot with anti-pMAPK1/3 and anti-MAPK1/3 antibodies (top) and anti-RIN1 and anti-tubulin antibodies (bottom). Band intensity was quantified using LI-COR Odyssey software. Phospho-MAPK1/3 signal intensities were normalized to total MAPK1/3 and RIN1 normalized to tubulin. Changes in expression or phosphorylation were calculated as fold-change compared to control vector (normalized to 1) and presented below each blot. The immunoblot shown is representative of three independent experiments. (**B**) K562 cells were treated with 1 μM imatinib (IM) for 1 and 4 hours at 37°C, then analyzed by immunoblot as in A. (**C**) K562 cells expressing control or RIN1 shRNA were analyzed by immunoblot as in A. The ratio of pMAPK/MAPK signal intensity is graphed. Results were averaged over five independent experiments. *p-value = 1x10^-4^

Downregulation of MAPK1 phosphorylation is a known consequence of imatinib inhibition of BCR-ABL1 in K562 cells, and it is required for imatinib-induced erythroid differentiation [26,27]. RIN1 depletion reduced the level of MAPK1/3 phosphorylation by 25 ± 4%. Conversely, RIN1 over-expression increased the level of this phosphorylation by 35 ± 3% (Fig 6A). BCR-ABL1 inhibition by imatinib rapidly decreased the MAPK1/3 phosphorylation signal (Fig 6B), suggesting that the RIN1 effect was mediated, at least in part, by ABL kinase activity. We next demonstrated that the decrease in MAPK1/3 phosphorylation in response to RIN1 silencing was strong and replicable (Fig 6C). While it has not been shown directly that RIN1::ABL binding is required for MAPK1/3 phosphorylation, growth factor stimulation that enhances MAPK1/3 phosphorylation also enhances RIN1-Y^36^ phosphorylation by ABL tyrosine kinases [28], which entails a physical interaction [29]. The responsiveness of MAPK1/3 phosphorylation status to RIN1 expression and BCR-ABL1 activity made it a useful surrogate for RIN1::ABL binding.

**Fig 7:**
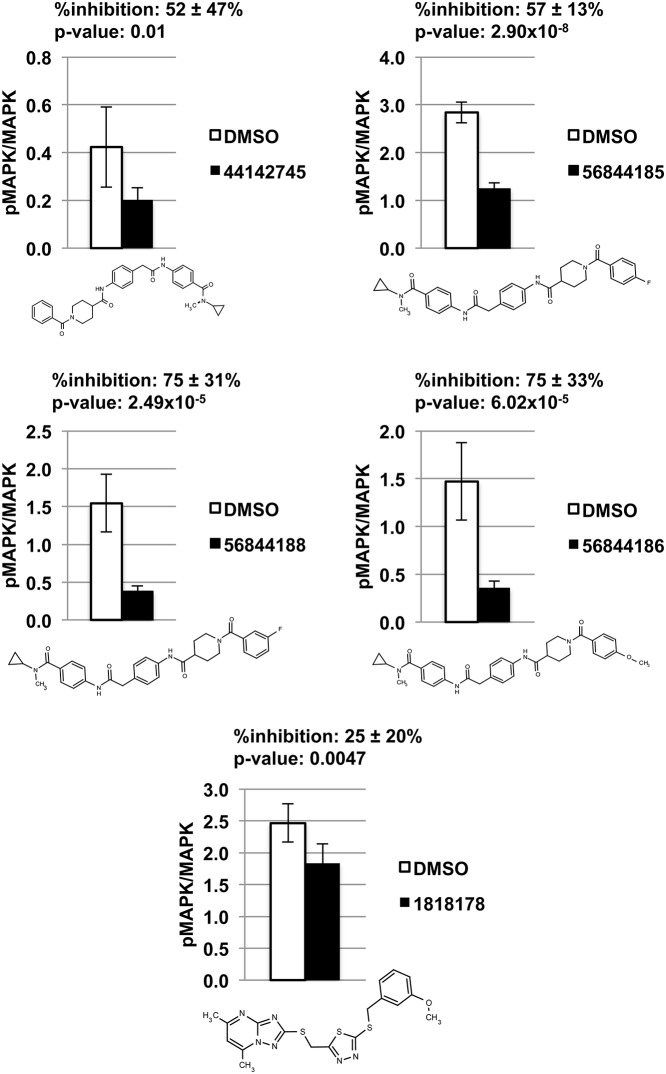
Five compounds significantly decrease MAPK1 phosphorylation in K562 cells. DMSO or compound was added to K562 cells at a final concentration of 1% or 10 μM, respectively, and incubated for 4 hours at 37°C before being lysed and analyzed by immunoblot with anti-pMAPK1/3 and anti-MAPK1/3. Each compound was tested with six biological replicates. Band intensity was quantified using LI-COR Odyssey, and the ratio of pMAPK/MAPK signal intensity is graphed for each compound and its DMSO control. Compounds are identified by their PubChem CID number.

Using this novel assay, we tested our 21 selected hits for their ability to disrupt RIN1-ABL signaling and identified five compounds in two structural classes that significantly decrease MAPK1/3 phosphorylation in K562 cells. One compound is a thiadiazole. The other four compounds are N-acetyl piperidine 4-carboxamides. Levels of inhibition ranged from 25–75% (Fig 7). These five lead compounds also decreased K562 proliferation in a dose-dependent manner (S2 Fig).

## Discussion

Despite the essential role of protein-protein interactions in cell signaling and disease, inhibition of these interactions *via* small molecules remains challenging. Contacts between proteins often involve large and flat interfaces with few apparent openings for high affinity small molecule binding [30]. Here we describe the design, validation and results of a robust TR-FRET based HTS to identify inhibitors of the RIN1::ABL1 protein-protein interaction that stimulates tyrosine kinase activity. Two design elements were crucial to the success of the assay. First, the use of the lanthanide element, terbium, contributed to an increased signal to noise ratio by allowing for delayed detection of the emission signal. Secondly, measurement of donor and acceptor emission and calculation of the FRET ratio decreased variability by normalizing for small differences in concentration.

The average Z’ and Z factors of the assay were consistently high, reflecting the large signal range and low well-to-well variability [31]. The standard deviation of Z’ was very small, reflecting good reproducibility of the assay over multiple plates and days. The difference between Z and Z’ revealed the effect of the compound library on the assay. For many plates, Z and Z’ values were very similar, because the compounds were inactive in the assay. However in some plates, large groups of structurally related compounds on the same plate were active in the assay, as reflected by large changes in the FRET ratio. This led to negative Z factors for some plates and contributed to a larger standard deviation for Z.

The primary screen identified 1,637 compounds, of which 708 were confirmed. Serendipitously, two of the hits provided further internal validation of the assay design. Theaflavin-3,3’-digallate was identified as a hit in the primary screen for its ability to decrease the FRET ratio. We also found that theaflavin-3,3’-digallate decreased phosphorylation of CRK and ABL *in vitro*, suggesting that it can function as a direct ABL kinase inhibitor. Its activity as a kinase inhibitor would be expected to destabilize the RIN1::ABL binding interaction by suppressing the RIN1 tyrosine phosphorylation needed for connecting with the ABL SH2 domain. This alone could explain the observed decrease in the FRET ratio. Moreover, theaflavin-3,3’-digallate may also bind RIN1’s proline-rich motif and block binding to the ABL SH3 domain, as other tea polyphenols have been shown to have affinity for proline rich regions [32,33].

The screen also identified an aminopyrimidine hit, 2-amino-6-[2-(4-methoxyphenyl)ethylamino]-1H-pyrimidin-4-one (CID 1532134), which shares core structural features with the allosteric BCR-ABL inhibitor GNF-1 and its analogue GNF-2, which are also aminopyrimidines (S3 Fig). GNF-2 binds to the kinase domain myristate pocket of BCR-ABL and thereby stabilizes the catalytically inactive conformation. And just as GNF-2 has no activity against native ABL1 [34,35], CID 1532134 had no activity against native ABL1 in our kinase assay, implying that it interferes with ABL activity but is not an active site competitor. Its structural similarity to GNF-2 suggests that CID 1532134 may inhibit RIN1::ABL complex formation allosterically by binding in the myristate pocket of ABL and stabilizing an inactive conformation.

In narrowing our list of hits, we sought to identify lead compounds that were effective in limiting BCR-ABL1 stimulation by RIN1 in cells. The K562 MAPK1/3 phosphorylation assay used here allowed for rapid and reproducible identification of compounds that disrupt RIN1-dependent BCR-ABL1 signaling. This also singled out compounds with favorable characteristics including stability, potency, target selectivity, solubility and cell permeability. Our cell assay identified five lead compounds: one thiadiazole and four structurally similar acyl piperidine carboxamides.

These five lead compounds are notable for their ability to alter BCR-ABL activity by targeting its interaction with the positive regulator RIN1. While BCR-ABL has been extensively targeted with direct kinase inhibitors, this is the first demonstration of selectively altering BCR-ABL kinase activity by interfering with a protein-protein interaction partner. Furthermore, we show that the compounds decrease MAPK1/3 phosphorylation in K562 cells, a downregulation that is required for imatinib-induced apoptosis and differentiation of leukemia cells [27].

Because the target selectivities of our lead compounds have not yet been established, we cannot exclude other possible mechanisms of p-MAPK1/3 reduction in the current study. Inhibition of upstream kinases, enhanced phosphatase activity or even direct binding to MAPK1/3 might inhibit MAPK1/3 phosphorylation. However, the results of our high throughput screen and the identification of lead compounds offer a novel tactic for targeting BCR-ABL oncoproteins, and provide a starting point for new molecular tools for understanding ABL tyrosine kinase regulation by RIN1 binding.

The manner in which these compounds bind and inhibit the formation of a RIN1::ABL complex is unclear. The acyl piperidine carboxamides are distinctly linear and extended molecules, however, perhaps making them well suited for interaction at a protein-protein interface. There was also strong evidence of a structure-activity relationship in this series (S4 Table). Several structural isomers of these hits, such as meta-substituted analogs in the two central phenyl rings and also 3-acyl piperidine analogues, were present in the screening collection. All such positional isomers were inactive in the TR-FRET screening assay (S4 Fig), suggesting that a large and elongated conformation is necessary for activity.

## Materials and Methods

### Plasmid construction and baculovirus production

The ABL1 amino terminus was amplified from AblWT1-531 (gift of Dr. John Kuriyan) and cloned into the pKS Bluescript vector. Insertion of the eGFP coding sequence with a His_6_ tag to the carboxy terminus created a 775 amino acid fusion (S5 Fig). This was subsequently moved into pFastBac1 for insect cell expression. The RIN1-SBP construct was generated by connecting a full-length human RIN1 coding sequence to the tandem affinity purification (TAP) tag from pSNSAP1 (PMID: 12574127). This generated a RIN1-SBP (streptavidin binding peptide)-TEV(protease cleavage site)-ProtA(immunoglobulin binding)-Flag(epitope) fusion orf that was cloned into pFastBac1 for insect cell expression. S5 Fig shows the amino acid sequence of each fusion protein construct. Baculovirus stocks were generated using the Life Technologies baculovirus expression system protocol. Briefly, pFastBac1 constructs were transformed into DH10Bac *E*. *coli* (Life Technologies) to generate recombinant bacmids, which were screened by blue/white selection. Recombinant bacmids were transfected into Sf9 cells using Cellfectin (Life Technologies). Medium containing passage 1 virus was harvested 72 hours post-transfection, clarified by centrifugation at 500*xg*, and stored at 4°C. Baculoviruses were then plaque purified and amplified using the same protocol.

### Purification of Recombinant Proteins

To express protein, 2.5x10^6^ Sf9 cells/mL were infected 1:10 with p3-p5 baculovirus. Cells were incubated at 27°C on 150 rpm shaker for 72 hours. Cells were collected and pelleted at 800*xg*. For ABL-eGFP-His_6_, cells were sonicated in 20 mM Tris-HCl pH 8, 100 mM NaCl and 5 mM imidazole. The lysate was clarified at 17,000*xg* for 30 minutes, and then incubated with Ni-NTA agarose (Qiagen) overnight. Beads were washed with 20 mM Tris-HCl pH 8, 250 mM NaCl, 10 mM imidazole and then eluted with 20 mM Tris-HCl pH 8, 250 mM NaCl, 10% glycerol and 100 mM imidazole, followed by 200 mM imidazole. Elutions were dialyzed in 50 mM Tris pH 8, 50 mM NaCl, 1 mM DTT and 10% glycerol. Protein concentration was determined by Bradford assay, and aliquots were frozen at -80°C until use.

For RIN1-SBP, protein was expressed as above. The cell pellet was sonicated in 20 mM Tris-HCl pH 8, 100 mM NaCl and 10% glycerol. The lysate was centrifuged at 17,000*xg* for 30 minutes, and incubated overnight with IgG sepharose (GE Healthcare Life Sciences). Beads were washed once with 50 mM Tris-HCl pH 8, 150 mM NaCl and. 05% Tween. They were then washed twice with TEV cleavage buffer: 50 mM Tris-HCl pH 8, 0.5 mM EDTA and 1 mM DTT. Beads were resuspended in TEV cleavage buffer, and incubated with TEV protease at 15°C overnight. After the supernatant containing RIN1-SBP was collected, beads were resuspended in 50 mM Tris pH 8, 2 M NaCl, 0.5 mM EDTA, 1 mM DTT, 1% NP-40 and 1% BRIJ35 and rotated for 1 hour at 4°C. This was done twice to wash the RIN1-SBP from the beads. Elutions were combined, dialyzed and aliquots frozen at -80°C until use.

### TR-FRET assay

For the TR-FRET assay, 75 nM purified ABL1-eGFP was pre-mixed with 100 nM RIN1-SBP and 2.5 nM Streptavidin-terbium (Life Technologies PV3966) in a kinase buffer consisting of 10 mM Tris pH 7.4, 100 mM NaCl, 10 mM MgCl_2_, 500 µM ATP, 1 mM DTT, 100 µM Na_3_VO_4_, 100 µM NaF and 100 µM BSA. For the UCLA MSSR screen, a Multidrop 384 was used to dispense 10 µL/well in a 384-well assay plate (Corning 3673). Compounds were added using Biomek FX, to a final concentration of 10 µM and 1% DMSO. The plates, with lids (Corning 3089), were then incubated for one hour at room temperature. After incubation, plates were read on a Perkin Elmer Victor 3 plate reader using 340 nm pulse, 100 µsec delay and 300 µsec window for signal. Emission filters were 520 ± 10 nm and 486 ± 5 nm.

The assay was further miniaturized to 1536 ultra-HTS format at TSRI, Florida and validated by comparing %inhibition and IC_50_ of a hit from the UCLA MSSR screen, CID 24512426. Details including data analysis methods and hit selection from the TSRI uHTS can be found at PubChem BioAssay AID 588664 (https://pubchem.ncbi.nlm.nih.gov/assay/assay.cgi?aid=588664).

#### Cheminformatics

Shared scaffolds of active compound families from the confirmation screen and CRC experiments were identified using a Maximum Common Substructure hierarchical clustering (ChemAxon LibraryMCS 5.10.2). LibraryMCS searches for the Maximum Common Substructures of a compound library in a hierarchical manner; where increasing molecular complexity of the shared substructure is represented down the dendrogram [36]. The physical properties (i.e. molecular mass, topological polar surface area, chiral atoms, H-bond acceptors/donors, ring count and rotatable bonds) of the compounds tested in dose-response format were calculated (ChemAxon Instant JChem 6.2.2).

#### Chemicals

The MLSMR library was provided by BioFocus DPI (South San Francisco, CA) through the NIH’s Roadmap Molecular Libraries Initiative. Details regarding compound selection for this library can be found online at http://mli.nih.gov/mli/compound-repository/mlsmr-compounds/. Briefly, this library is a highly diversified collection of small molecules (more than 50% in the molecular weight range 350–410 g/mol) and is comprised of both synthetic and natural products, from either commercial or academic sources, that can be grouped into the 4 following categories: (1) specialty sets of known bioactive compounds such as drugs and toxins (0.65%), (2) focused libraries aimed at specific target classes (2.85%), (3) non-commercial sources (7.4%) and (4) diversity sets covering a large area of the chemical space (89.1%).

### *In vitro* kinase assay for direct ABL inhibitors

The kinase assay to identify direct ABL kinase inhibitors from UCLA MSSR and TSRI hits was performed as described in PubChem BioAssay AID 624303 (https://pubchem.ncbi.nlm.nih.gov/assay/assay.cgi?aid=624303). Purified His_6_-CrkII was expressed and purified as previously described [19].

### Mass spectrometry and phosphopeptide identification of RIN1-dependent BCR-ABL1 substrates

K562 [37] cells were cultured in RPMI with 10% FBS and 1% Pen/Strep. K562 with control and RIN1 shRNA were previously described [20]. K562 cells expressing control shRNA or RIN1-shRNA were grown, processed and analyzed by MS twice independently. Cells were cultured in the presence of antibiotic during expansion before MS analysis, and were lysed by sonication in urea buffer (8 M urea, 50 mM Tris-HCl pH 7.5, 1 mM vanadate). Phosphotyrosine peptide were immunoprecipitated with anti-phosphotyrosine antibodies (Millipore, clone 4G10) using 2x10^8^ cells as previously described [38].

Phosphorylated peptides were analyzed by LC-MS/MS using an autosampler coupled with Nano2DLC pump (Eksigent) and LTQ-Orbitrap (Thermo Fisher Scientific). The samples were loaded onto an analytical column (10 cm ×75 μm i.d.) packed with 5 μm Integrafit Proteopep2 300 Å C18 (New Objective). Peptides were eluted into the mass spectrometer using a HPLC gradient of 5−40% Buffer B in 45 min followed by a quick gradient of 40−90% Buffer B in 10 min, where Buffer A contains 0.1% formic acid in water and Buffer B contains 0.1% formic acid in acetonitrile (Ultima Gold, Fisher Scientific). Mass spectra were collected in positive ion mode using the Orbitrap for parent mass determination and the LTQ for data-dependent MS/MS acquisition of the top five most abundant peptides. Each sample was analyzed twice (replicate runs), and in each run, one-half of the sample was injected.

MS/MS fragmentation spectra were searched with SEQUEST (Version v.27, rev. 12, Thermo Fisher Scientific) against a database containing the human International Protein Index (IPI) protein database (ftp://ftp.ebi.ac.uk). Search parameters included carboxyamidomethylation of cysteine as static modification. Dynamic modifications included phosphorylation on tyrosine, and oxidation on methionine. Results derived from database searching were filtered using the following criteria: Xcorr >1.0(+1), 1.5(+2), 2(+3); peptide probability score <0.001; dCn >0.1; and mass accuracy <5 ppm (parts per million) with Bioworks version 3.2 (Thermo Electron Corp.). We estimated the false-positive rate of sequence assignments at 0.5% on the basis of a composite target-reversed decoy database search strategy [39]. The Ascore algorithm was used to more accurately localize the phosphate on the peptide (http://ascore.med.harvard.edu) [40].

As is common in data-dependent MS2 fragmentation sequencing, some peptides identified by sequencing in one sample may not be sequenced or identified in another sample even if the peak is present. Peptide peaks sequenced in some samples but not in others were located in the remaining samples by aligning the chromatogram elution profiles by means of a dynamic time warping algorithm [41]. An extended explanation of the strategy used in this work, and example performance results, can be found in the supporting information of Zimman *et al* [38].

### Antibodies and Immunoblotting

The following antibodies were used: monoclonal rabbit-anti-pERK1 pY^204^/ERK2 pY^187^ 1:500 (Epitomics, 2219–1), monoclonal mouse-anti-ERK1/2 1:5000 (BD, 610123), monoclonal mouse-anti-RIN1 1:500 (Colicelli lab, clone C9E11, Abpro), polyclonal rabbit-anti-ABL 1:1000 (Santa Cruz Biotechnology, sc-131), monoclonal mouse-anti-BCR 1:1000 (Santa Cruz Biotechnology, sc-48422), mouse-anti-tubulin 1:5000 (Sigma-Aldrich, T6074), goat-anti-rabbit-IRDye 800 1:5000 (Li-Cor Biosciences, 926–32211) and goat-anti-mouse-IRDye 680 1:5000 (Li-Cor Biosciences, 926–32220).

Membranes were scanned and signal intensity quantified using a Li-Cor Odyssey scanner. To quantify RIN1 expression, signal intensities were first normalized to the housekeeping gene, tubulin. Phospho-MAPK1/3 signal intensities were normalized to total MAPK1/3. Changes in protein expression or phosphorylation were calculated as fold-change compared to control vector.

### pMAPK in K562

K562 with vector or RIN1 overexpression lentivirus were previously described [20]. To analyze MAPK1 phosphorylation, confluent K562 cells were lysed in NP-40 buffer and immunoblotted. To test the effect of imatinib on MAPK phosphorylation, K562 cells were seeded 5x10^5^/well in a 12-well plate and incubated overnight at 37°C and 5% CO_2_. Cells were treated with 1 µM imatinib for 1 or 4 hours. Cells from each well were harvested, lysed in 400 µL NP-40 and immunoblotted.

To test each compound, 5x10^5^ K562 cells were plated on a 10 cm tissue culture plate and incubated for 3 days. Cells were harvested and the volume brought to 12 mLs with fresh medium. 1 mL of the cells was distributed into each well of a 12-well tissue culture plate. In 6 of the wells, 10 µL of 1 mM compound was added per well. 10 µL of DMSO was added to the remaining wells and mixed by pipetting up and down. Cells were incubated at 37°C and 5% CO_2_ for 4 hours. After incubation, cells were harvested from each well, lysed in 150 µL of RIPA buffer and analyzed as above.

### K562 Proliferation Assays

K562 cells were plated 3x10^4^ cells/well in 96-well plates. Cells were treated with DMSO or test compound in dose-response (100, 50, 25, 12.5, 6.25, 3.125 μM) to a final DMSO concentration of 1%. After 48 hours of incubation at 37°C, cell proliferation was assessed by MTS assay (Promega CellTiter 96 AQueous One Solution) according to the manufacturer’s protocol. Absorbance at 490nm was read on a PerkinElmer Victor3 plate reader. Experiments were performed in triplicate.

### Statistical Analysis

Two-tailed, equal variance Student’s *t* tests were used to analyze the data from TR-FRET and K562 cell-based assays. K562 phosphoprofiling data were analyzed by two-tailed, equal variance Student’s *t* tests, with and without Bonferroni correction.

## Supporting Information

S1 Fig Scaffold clustering of selected hits. Pyridone/pyrimidines, acyl piperidine carboxamides and heterocyclic amides are the largest clusters, with 8, 9 and 17 members, respectively. Three compounds are macrocycles, four are tetrazoles and we identified one indole alkaloid (CID 44601827) and one thiadiazole (CID 1818178). Representative compounds are shown for groups 1–5 and their PubChem CID numbers are as follows: (1) 3607724, (2) 44142745, (3) 24686095, (4) 44502732, (5) 51360358.
(PDF)

S2 Fig The five lead compounds decrease K562 proliferation in a dose-dependent manner. K562 cells were treated with DMSO or test compound in dose-response. After 48 hours of incubation at 37°C, cell proliferation was assessed by MTS assay. Growth in the presence of test compound was normalized to DMSO-treated K562 cells and results are presented as % of growth relative to control. Experiments were performed in triplicate.
(PDF)

S3 Fig CID 1532134 is structurally similar to known allosteric BCR-ABL kinase inhibitors GNF-1 and GNF-2. (PDF)

S4 Fig Acyl piperidine carboxamide structure-activity relationship. (PDF)

S5 Fig ABL-eGFP and RIN1-TAP protein sequences. (PDF)

S1 Table Confirmed hits from UCLA MSSR screen. (XLSX)

S2 Table 21 hits selected for cell-based assay. (XLSX)

S3 Table Phosphotyrosine peptides from K562 ctrl vs. K562 RIN1 knockdown. (XLSX)

S4 Table N-acyl piperidine-4-carboxamide Series SAR table. (XLSX)
