# Supplementary figures and images for: Identification of Small Molecules that Disrupt Signaling between ABL and Its Positive Regulator RIN1
Source: PLoS One. 2015 Mar 26;10(3):e0121833. doi: 10.1371/journal.pone.0121833 (PMC4374917; doi:10.1371/journal.pone.0121833)

**S1 Figure. Scaffold clustering of selected hits.**

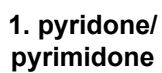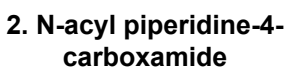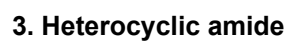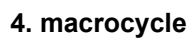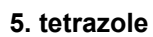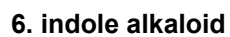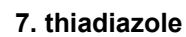

Supplement: S1 Fig — Pyridone/pyrimidines, acyl piperidine carboxamides and heterocyclic amides are the largest clusters, with 8, 9 and 17 members, respectively. Three compounds are macrocycles, four are tetrazoles and we identified one indole alkaloid (CID 44601827) and one thiadiazole (CID 1818178). Representative compounds are shown for groups 1–5 and their PubChem CID numbers are as follows: (1) 3607724, (2) 44142745, (3) 24686095, (4) 44502732, (5) 51360358. (PDF) [file pone.0121833.s001.pdf]

**S2 Figure. The five lead compounds decrease K562 proliferation in a dose-dependent manner.**

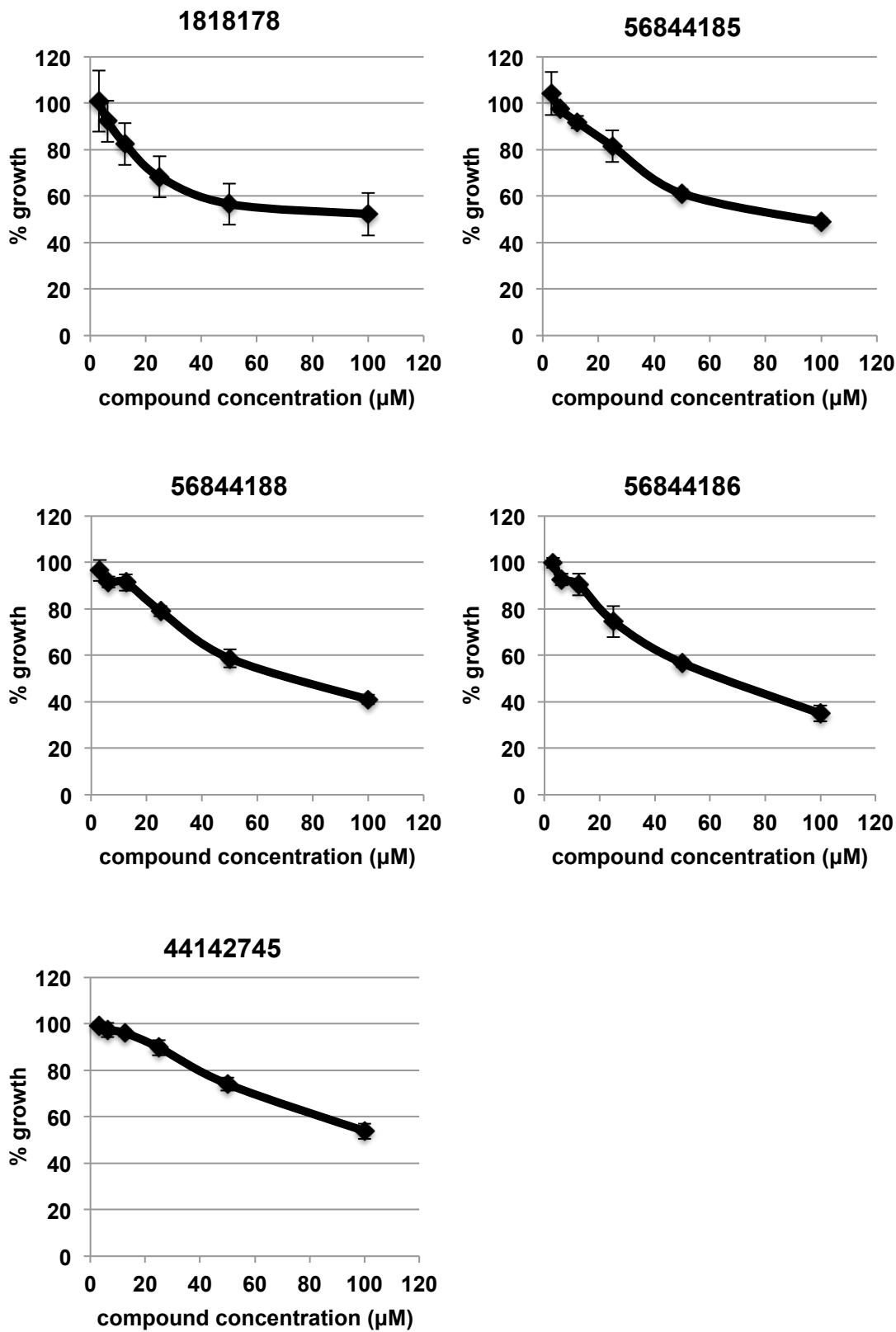

Supplement: S2 Fig — K562 cells were treated with DMSO or test compound in dose-response. After 48 hours of incubation at 37°C, cell proliferation was assessed by MTS assay. Growth in the presence of test compound was normalized to DMSO-treated K562 cells and results are presented as % of growth relative to control. Experiments were performed in triplicate. (PDF) [file pone.0121833.s002.pdf]

**S4 Figure. Acyl piperidine carboxamide structure-activity relationship.**

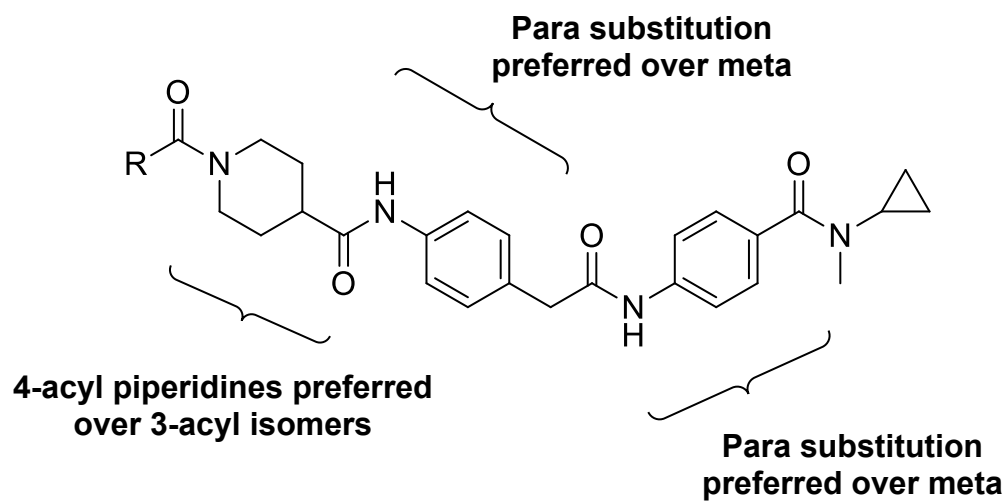

Supplement: S4 Fig — (PDF) [file pone.0121833.s004.pdf]
