# Supplementary material for: Identification of Small Molecules that Disrupt Signaling between ABL and Its Positive Regulator RIN1
Source: PLoS One. 2015 Mar 26;10(3):e0121833. doi: 10.1371/journal.pone.0121833 (PMC4374917; doi:10.1371/journal.pone.0121833)
Supplement: S3 Fig — (PDF) [file pone.0121833.s003.pdf]

**S3 Figure. CID 1532134 is structurally similar to known allosteric BCR-ABL kinase inhibitors GNF-1 and GNF-2.**

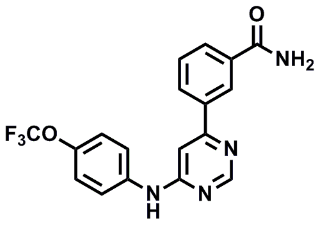

**GNF-2**

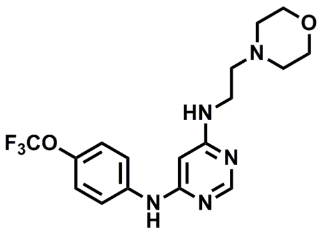

**GNF-1**

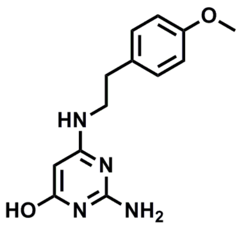

**CID 1532134**
