# Supplementary material for: Identification of Small Molecules that Disrupt Signaling between ABL and Its Positive Regulator RIN1
Source: PLoS One. 2015 Mar 26;10(3):e0121833. doi: 10.1371/journal.pone.0121833 (PMC4374917; doi:10.1371/journal.pone.0121833)
Supplement: S5 Fig — (PDF) [file pone.0121833.s005.pdf]

S5 Figure. ABL-eGFP and RIN1-TAP protein sequences.

ABL1[531]**-GFP MW aprox. 88 kDa**
MGQQPGKVLGDQRRPSLPALHFIKGAGKKESSRHGGPHCNVFVEHEALQRPVASDFE
PQGLSEAARWNSKENLLAGPSENDPNLFVALYDFVASGDNTLSITKGEKLRVLGYNHNG
EWCEAQTKNGQGWVPSNYITPVNSLEKHSWYHGPVSRNAAEYLLSSGINGSFLVRESE
SSPGQRSISLRYEGRVYHYRINTASDGKLYVSSESRFNTLAELVHHHSTVADGLITTLHYP
APKRNKPTVYGVSPNYDKWEMERTDITMKHKLGGGQYGEVYEGVWKKYSLTVAVKTLK
EDTMEVEEFLKEAAVMKEIKHPNLVQLLGVCTREPPFYIITEFMTYGNLLDYLRECNRQE
VNAVVLLYMATQISSAMEYLEKKNFIHRDLAARNCLVGENHLVKVADFGLSRLMTGDTYT
AHAGAKFPIKWTAPESLAYNKFSIKSDVWAFGVLLWEIATYGMSPYPGIDLSQVYELLEK
DYRMERPEGCPEKVYELMRACWQWNPSDRPSFAEIHQAFETMFQESSISDEVEKELG
KMSKGEELFTGVVPILVELDGDVNGHKFSVSGEGEGDATYGKLTLKFICTTGKLPVPWP
TLVTTLTYGVQCFSRYPDHMKQHDFFKSAMPEGYVQERTIFFKDDGNYKTRAEVKFEG
DTLVNRIELKGIDFKEDGNILGHKLEYNYNSHNVYIMADKQKNGIKVNFKIRHNIEDGSVQ
LADHYQQNTPIGDGPVLLPDNHYLSTQSALSKDPNEKRDHMVLLEFVTAAGITHGMDEL
YKHHHHHH

RIN1-TAP
**MESPGESGAGSPGAPSPSSFTTGHLAREKPAQDPLYDVPNASGGQAGGPQRPGRVV
SLRERLLLTRPVWLQLQANAAAALHMLRTEPPGTFLVRKSNTRQCQALCMRLPEASG
PSFVSSHYILESPGGVSLEGSELMFPDLVQLICAYCHTRDILLLPLQLPRAIHHAATHKE
LEAISHLGIEFWSSSLNIKAQRGPAGGPVLPQLKARSPQELDQGTGAALCFFNPLFPG
DLGPTKREKFKRSFKVRVSTETSSPLSPPAVPPPPVPVLPGAVPSQTERLPPCQLLRR
ESSVGYRVPAGSGPSLPPMPSLQEVDCGSPSSSEEEGVPGSRGSPATSPHLGRRRPL
LRSMSAAFCSLLAPERQVGRAAAALMQDRHTAAGQLVQDLLTQVRDGQRPQELEGIR
QALSRARAMLSAELGPEKLVSPKRLEHVLEKSLHCSVLKPLRPILAARLRRRLAADGS
LGRLAEGLRLARAQGPGAFGSHLSLPSPVELEQVRQKLLQLVRTYSPSAQVKRLLQA
CKLLYMALRTQEGEGSGADGFLPLLSLVLAHCDLPELLLEAEYMSELLEPSLLTGEGG
YYLTSLSASLALLSGLGQAHTLPLSPVQELRRSLSLWEQRRLPATHCFQHLLRVAYQD
PSSGCTSKTLAVPPEASIATLNQLCATKFRVTQPNTFGLFLYKEQGYHRLPPGALAHRL
PTTGYLVYRRAEWPETQGAVTEEEGSGQSEARSRGEEQGCQGDGDAGVKASPRDIR
EQSETTAEGGQGQAQEGPAQPGEPEAEGSRAAEE**SRMDEKTTGWRGGHVVEGLAG
ELEQLRARLEHHPQGQREPSGGCKLGLLVPRGSASENLYFQGELKTAALAQHDEAVDN
KFNKEQQNAFYEILHLPNLNEEQRNAFIQSLKDDPSQSANLLAEAKKLNDAQAPKVDNK
FNKEQQNAFYEILHLPNLNEEQRNAFIQSLKDDPSQSANLLAEAKKLNGAQAPKVDANS
AGKSTQL*DYKDDDDK*

EGFP
SBP
QG = TEV cleavage
ProtA
FLAG epitope tag
